# Mechanical Behavior of One-Piece and Two-Piece Tapered Prosthetic Abutments with 11.5 and 16 Degrees

**DOI:** 10.3390/healthcare11121775

**Published:** 2023-06-16

**Authors:** Karine Regina Tolesano Loureiro, Marcos Boaventura de Moura, Guilherme José Pimentel Lopes de Oliveira, Veridiana Resende Novais, Karla Zancopé, Paulo Cézar Simamoto Júnior

**Affiliations:** 1 Department of Occlusion, Fixed Prosthodontics and Dental Materials, School of Dentistry, Federal University of Uberlandia, Uberlandia 38405-320, MG, Brazil; karine.t.loureiro@gmail.com (K.R.T.L.); psimamoto@gmail.com (P.C.S.J.); 2 Independent Researcher, Curitiba 80820-520, PR, Brazil; boaventura.mm@hotmail.com; 3 Department of Periodontology and Implantology, School of Dentistry, Federal University of Uberlandia, Uberlandia 38405-320, MG, Brazil; guilherme.lopesoliveira@ufu.br; 4 Department of Operative Dentistry and Dental Materials, School of Dentistry, Federal University of Uberlandia, Uberlandia 38405-320, MG, Brazil; veridianaresende@hotmail.com

**Keywords:** biomechanics, finite element analysis, fatigue, implantology, pull-out

## Abstract

The objective of this study is to examine the mechanical behavior of two-piece abutments (Morse taper with 16° internal angulation and Morse taper with 11.5° internal angulation) before and after cyclic fatigue testing, following ISO 14801:2016 guidelines. The specimens were divided into three groups: a modified Morse taper with a taper angle of 16° (GM group), a conventional Morse taper (taper angle of 11.5° deg) with a two-piece (CMt group), and one-piece abutments (CMo group). Each experimental group was formed by ten implants and ten abutments (*n* = 10) for a total of 30 specimens (*n* = 30). The abutments were tightened and loosened, and a fatigue test was applied with 15 Hz and 5 × 10^6^ cycles. Subsequently, the abutments were loosened, and a pull-out test was performed on the CMt group. Finite element analysis (FEA) was conducted on stress concentration regions. The statistical analysis of the loosening test was performed using two-way ANOVA and Tukey’s tests (*p* < 0.05) to compare screw loosening within each group and between the groups with and without mechanical fatigue. Significant differences were found among the three groups in the loosening test when analyzing the values with and without fatigue (*p* < 0.001) within each group. When the groups were compared with each other, there was also a significant difference between them (*p* < 0.001), except between groups GM and CMt without fatigue (*p* = 0.840). In the pull-out test of the CMt group, the sample exhibited frictional locking only after fatigue (mean = 94.2 N). The FEA demonstrated a varied stress distribution in all groups. The stress was found to be more concentrated in the upper third and middle third regions of the implant, as well as in the opposite region of the load application for all three groups. Although the CMo group showed lower rates of loosening, it displayed a poorer stress distribution in comparison to the GM and CMt groups. On the other hand, the CMt group exhibited a satisfactory frictional lock after undergoing the fatigue tests.

## 1. Introduction

One of the important factors that determine the stability and fixation of internal tapered implant–abutment joints is the frictional resistance generated by the contact between the tapered regions of the abutment and implant coupling. This mechanism relies on friction rather than the screw for support [1]. Mathematical equations and finite element (FE) models have demonstrated that over 86% of the tightening torque and over 98% of the relaxation torque are counterbalanced by the tapered connection of these systems [1]. However, the application of occlusal load is a potential factor that may cause the retention screw to loosen and the abutment to shift position [2,3].

Usually, torque is applied to the abutment screw using a torque wrench to properly secure the abutment to the implant. This tightening creates an axial tension on the surface of the screw that is in contact with the adjacent material. This is known as preload. The magnitude of preload is of great importance for the success of dental implants. Most two-piece dental implants fail due to screw loosening [4]. When the screw is tightened against a material, it allows the screw to distribute the force through the material so that the screw itself retains only a portion of the load. This means that the screw can withstand a significantly higher load when the correct amount of tension is applied. Therefore, it is essential to apply the preload recommended by the manufacturer to the screw. Adding the recommended preload can help a screw find a more favorable combination of stress amplitude and mean stress, resulting in increased fatigue life [4].

The geometry of the implant–abutment interface seems to be an influencing factor for the transmission of stress around the implant [5,6,7,8]. In a study using Morse taper implants with solid one-piece abutments, the relationship between crown size and implant size was evaluated, revealing that the anterior upper abutments exhibited a higher tendency for loosening. In the posterior regions, the abutments either fractured or became loose [9]. The use of a tapered connection and abutment with an integrated screw (two-piece) is being adopted, where the screw and a tapered adjustment are used simultaneously to provide mechanical stability [8]. This type of joint offers high resistance to screw-loosening torque and abutment [10].

The long-term success of implant-supported restorations greatly depends on the stability of the implant–abutment assembly [5,11,12,13]. Internally tapered connections have been recognized for their favorable mechanical stability [13]. However, further studies are necessary, especially considering that different geometries of both implants and abutments are frequently introduced in the market simultaneously [11,12]. Therefore, it is crucial to investigate various aspects of these new implant–abutment configurations, including mechanical resistance and stress distribution [13,14]. This emphasizes the importance of studying novel implant geometries and their implications.

Different features are incorporated into the design of dental implants to achieve satisfactory mechanical performance. A different configuration of the Morse cone was evaluated in this study, presenting an internal conical portion angled at 16° (half-angle of 8°). The two-piece universal abutment has a self-removing transfixing screw and does not rely on mechanical friction locking. The other configurations of the Morse cone evaluated in this study had an internal conical portion angled at 11.5° (half an angle of 5.75°). The universal abutments for this implant can be one-piece or two-piece, with the two-piece abutment equipped with a transfixing screw that maintains the frictional fit between the inner walls of the implant and the abutment even after it has been unscrewed.

**Figure 1:**
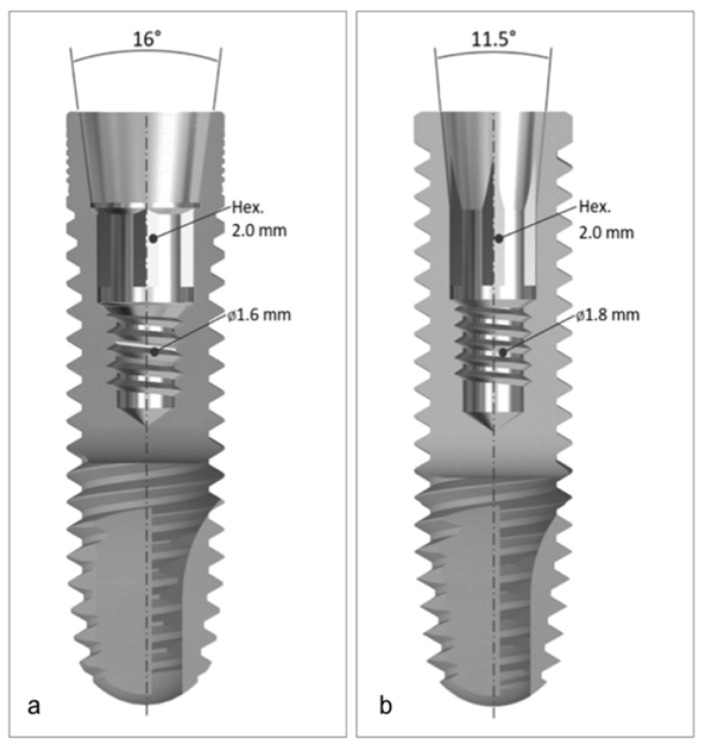
(**a**) Titamax GM Neodent Implant—16 degrees taper angle (half-angle of 8); (**b**) Titamax Cortical CM Neodent Implant—11.5 degrees taper angle (half-angle of 5.75).

**Figure 2:**
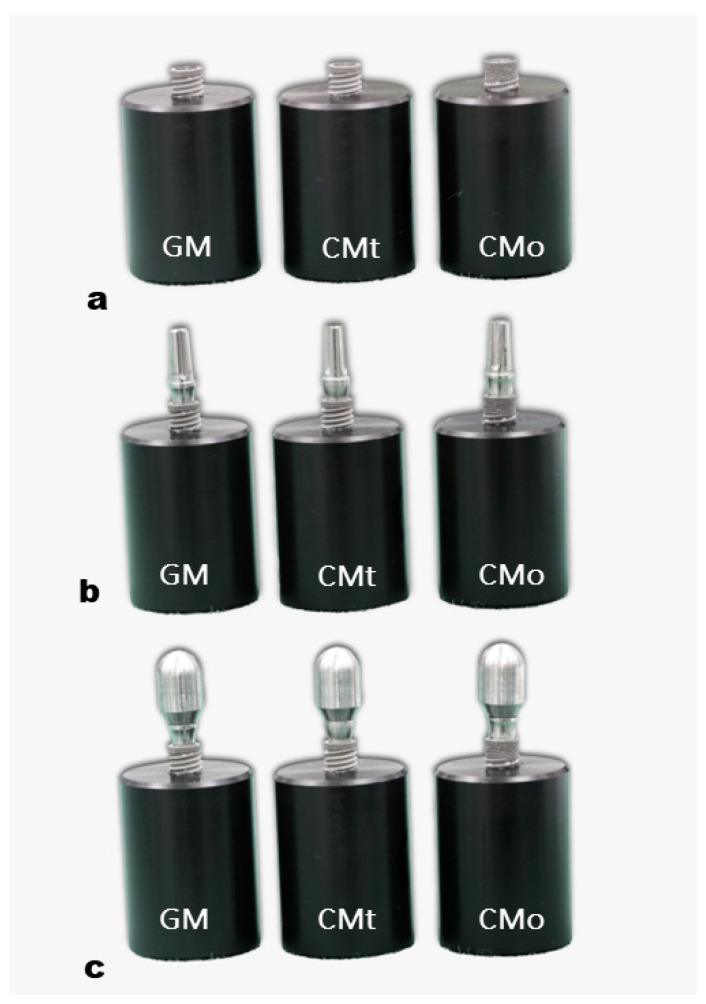
Types of implant–abutment connections used in the study. (**a**) GM, CMt, and CMo implants were installed in a cylindrical block made of polyacetal; (**b**) GM prosthetic abutments with a two-piece abutment design featuring a 16° taper, CMt prosthetic abutments with a two-piece abutment design featuring an 11.5° taper, and CMo prosthetic abutments with a one-piece abutment design featuring an 11.5° taper; (**c**) prosthetic abutments were covered with a semi-spherical rigid body.

The objective of this study is to evaluate the mechanical behavior of two-piece abutments (Ti-6Al-4V) with different internal angulations (16° and 11.5°) before and after cyclic fatigue testing, following the ISO 14801:2016 guidelines. Two types of internal tapered implants (Neodent) were used, one with a 16° angulation (Ti-6Al-4V) and the other with an 11.5° angulation (Ti-6Al-7Nb) (Figure 1). The specimens were divided into three groups: GM group (modified Morse taper with a 16° angulation), CMt group (conventional Morse taper with an 11.5° angulation and two-piece abutments), and CMo group (conventional Morse taper with an 11.5° angulation and one-piece abutments) (Figure 2) (Table 1). Each group consisted of 10 implants and 10 abutments, resulting in a total of 30 specimens (*n* = 30). The pull-out test was conducted to evaluate the tensile strength of the Morse taper implants and one-piece abutments with an 11.5 degrees taper angle. Finite element analysis (FEA) was performed to assess the stress levels in the mechanically tested prosthetic abutments after cyclic fatigue. The study tested two hypotheses: (1) there would be no significant difference in the screw loosening values among the tested abutments, and (2) the stress levels in the implant–abutment assemblies of the three groups would be similar after simulating the fatigue test.

## 2. Materials and Methods

### 2.1. Specimens Preparation

Thirty implants with a Morse taper design (10 Titamax GM and 20 Titamax CM Cortical, 3.75 mm diameter/13 mm length) (Neodent, Curitiba, PR, Brazil) and 30 prosthetic abutments (10 Universal Abutment GM exact—“two-piece”, 20 Universal Abutment CM—“two-piece” and 20 Universal Abutment CM—“one-piece”, 3.3 mm diameter/6 mm crown height/2.5 mm gingival height (Neodent, Curitiba, PR, Brazil) were connected using three different tightening forces: 20 Ncm (GM), 15 Ncm (CMt), and 32 Ncm (CMo); manufacture’s recommended torques. Each experimental group was formed by 10 implants and 10 abutments (*n* = 10) for a total of 30 specimens (*n* = 30) (Table 1).

All implants were placed in a cylindrical block made of polyacetal, leaving 3 mm of exposure to these implants, as recommended by ISO 14801 (Figure 2a).

The sample implants were placed on a rigid base used for 30 degrees angle anchorage, 3.0 mm above the bone (simulating high bone resorption) [15,16]. A digital prosthetic ratchet (TQ-680, Instrutherm, Ltd.a., São Paulo, Brazil) was used for tightening the prosthetic abutments to the implants [13]. The abutments of the three groups were tightened to the manufacturer-recommended torques (GM, 20 N cm; CMt, 15 N cm, CMo, 32 N cm), and after 5 min, the abutments were loosened, and the loosening values were recorded. (Figure 2b). Then, another tightening was performed for the mechanical fatigue test.

### 2.2. Fatigue Test

**Figure 3:**
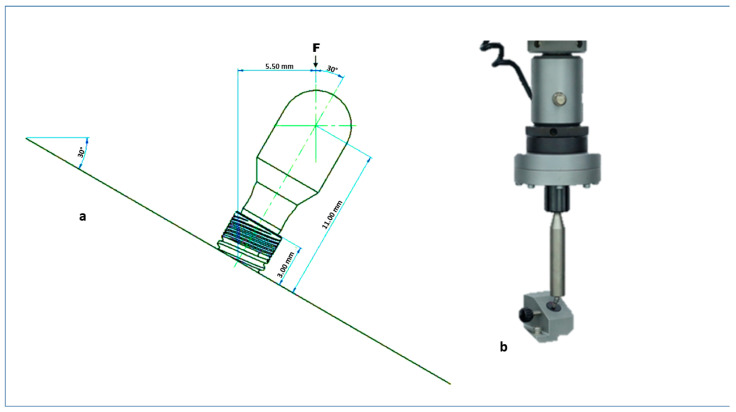
(**a**) Schematic of fatigue test. (**b**) device with a flat surface perpendicular to the loading direction (Instron E3000 with a capacity of 3 kN; Instron, Norwood, MA, USA).

The fatigue loading test was performed according to ISO 14801:2016 [8,15]. The coronal section end of the prosthetic abutment was covered with a semi-spherical rigid body (Figure 2c), the center of which coincided with the center of the free longitudinal axis and was anchored at 11.0 ± 0.5 mm from the bone level (measured on a line parallel to the longitudinal axis of the implant) (Figure 3).

To simulate a worst-case condition, the specimens were inclined at a 30° angle from the vertical axis to apply vertical loads, combining bending and torque moments on the internal tapered connection. A semi-spherical rigid body was used on the cementable part of the abutments, with its center coinciding with the center of the free longitudinal axis and anchored at a distance of 11.0 ± 0.5 mm from the bone level, measured along a line parallel to the implant’s longitudinal axis (Figure 3b).

The loading force was applied to the surface of the hemispherical body using a device with a flat surface perpendicular to the loading direction (Figure 1b). The device was not constrained in the transverse direction of the load to prevent a reduction in the generated bending moment’s magnitude [15,16].

A static loading test was conducted at a speed of 1.0 mm/min to determine the maximum load [17]. A flexural load was applied to the implant–abutment assemblies at a rate of 0.5 mm/s [18]. The bending load acquired by the load cell was plotted on a load versus displacement curve. A computer associated with the machine was programmed to obtain the mechanical behavior of the specimen using a load cell and a load sensor. The test machine was programmed to stop the force test process for a greater displacement of 5.0 mm or an abrupt decrease in the strength of the tested material. Subsequently, dynamic loading was applied starting from the maximum load obtained in the static loading test. The dynamic fatigue test was performed using a wear simulator (Instron E3000 with a capacity of 3 kN; Instron, Norwood, MA, USA) with a loading frequency of 15 Hz and 5 × 10^6^ cycles, simulating a clinical estimate of five to six years of function [15,16,19,20]. Afterward, the abutments were loosened, and the loosening values were recorded. 

### 2.3. Pull-Out Test

**Figure 4:**
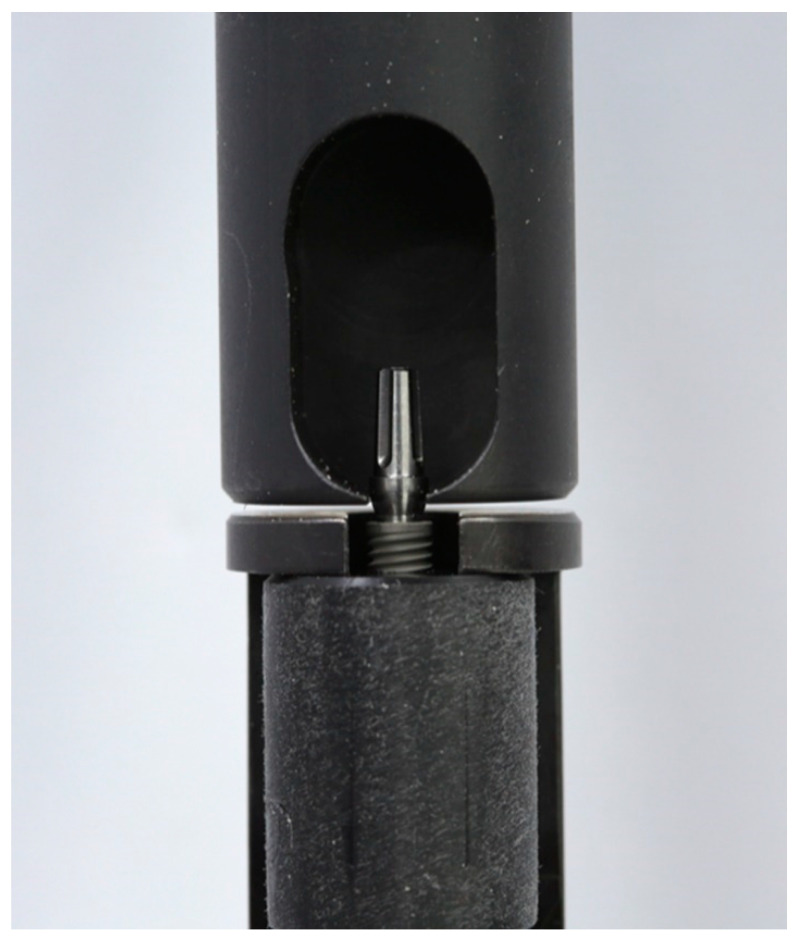
Assembly implant–abutment placed for the pull-out test.

The pull-out test was performed to evaluate the tensile strength of the Morse taper implants and abutments (CMt Group) with an 11.5 degrees taper angle at the following two moments: after initial screw loosening and after mechanical fatigue. The tensile strength of the GM and CMo group implants and abutments has not been tested because the GM group (16 degrees taper angle) has abutment self-removal and is easily removed as soon as the screw is loosened; the abutments of the CMo group (11.5 degrees taper angle) are one-piece, with an apical threaded portion [21]. The implant–abutment assemblies were placed in a mechanical testing machine (MultiTest 2.5 XT; Mecmesin, Sterling, VA, USA) (Figure 4).

The tensile strength (N) required for abutment removal was measured at a speed of 5 mm/min [16,17], and the data obtained were analyzed with computer software (MultiTest 2.5 XT, www.mecmesin.com; Mecmesin, Sterling, VA, USA) [16]. The pull-out test was described by the mean and standard deviation (SD) before and after mechanical fatigue.

### 2.4. Finite Element Analysis (FEA)

Computer-aided design (CAD) images obtained from the manufacturer were developed with Autodesk Inventor Professional 2013 (San Rafael, CA, USA) of the same components used for mechanical testing and were imported into the FE software (Inventor® versão 11.3.2). A layer of element friction between the implant and prosthetic abutment was inserted (0.5 coefficient of friction) [21,22]. An oblique force of 50 N determined by static fracture testing at 30 degrees from the axis of the implants was applied for each tested group in the numerical analysis. Torque relates to the resulting axial preload in the abutment, similar to the screw preload due to friction forces, which were applied between the thread and screw head [13]. The relationship between torque and axial preload is influenced by the friction forces acting between the screw threads and the screw head. When torque is applied to tighten a screw, the friction forces between the threads and head resist the rotation, resulting in an axial force being exerted on the abutment. This axial force is commonly referred to as the preload. The determined axial preload for each group (200 N for GM, 150 N for CMt, and 320 N for CMo) was introduced into the model with a layer of elements between the screw body for GM and CMt and the abutment for CMo.

**Figure 5:**
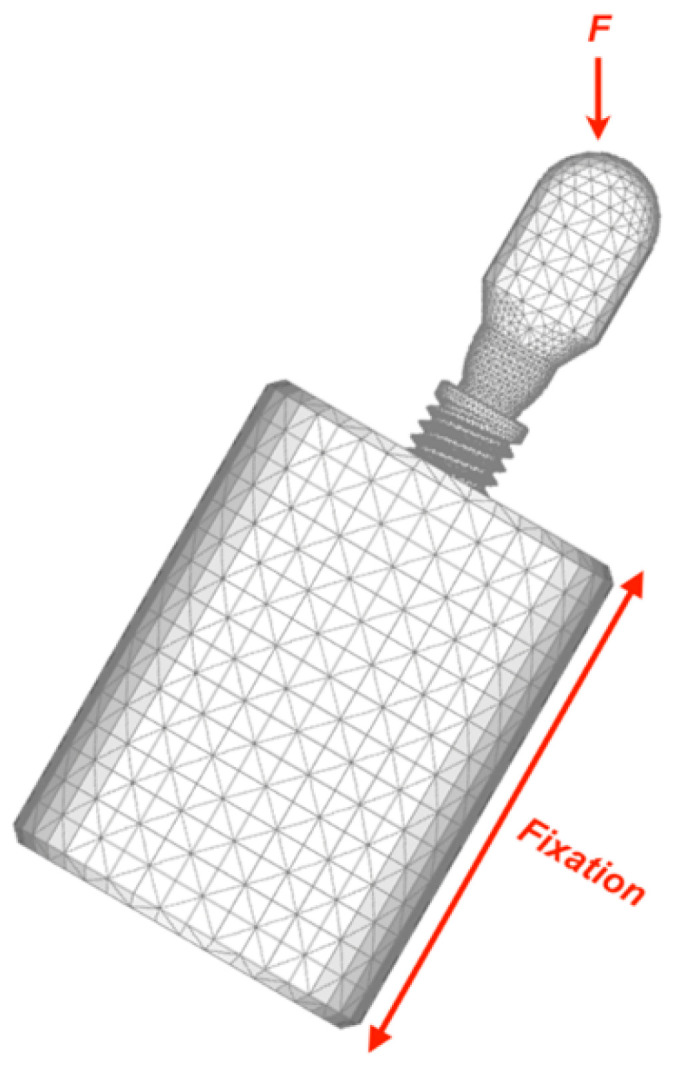
A finite element mesh that simulates the mechanical testing using a vertical load applied to specimens inclined 30 degrees from the vertical axis was developed with Autodesk Inventor Professional 2013 (San Rafael, CA, USA).

Finite element analysis (FEA) was used to predict the mechanical performance of dental implants, specifically to analyze critical regions between different types of components and prosthetic connections [23]. The FEMAP v10.2.0 (Siemens PLM Software, Plano, TX, USA) software was used for the pre- and post-processing phases. In the pre-processing stage, meshes were generated with tetrahedral parabolic solid elements. The mechanical properties of each simulated material were attributed to the meshes provided by the manufacturer (Table 2). All materials were considered isotropic, homogeneous, and linearly elastic for the three different types of implant–abutment assemblies used in this study to simulate the mechanical test (Figure 5). 

The final FE models after convergence testing were a tridimensional mesh incorporating tetrahedra (GM: 91,842 elements and 416,252 nodes; CMt: 72,128 elements and 330,251 nodes; CMo: 72,992 elements and 339,460 nodes).

### 2.5. Statistical Analysis

Statistical analysis of the loosening test was performed with two-way ANOVA and Tukey tests (*p* < 0.05) to compare screw loosening within each group and between groups with and without mechanical fatigue. To perform all loosening analyses, the statistical software Sigma Plot (version 12.0; Systat Software Inc., San Jose, CA, USA) was used.

## 3. Results

No specimen failed after fatigue testing. The results of the loosening test were presented by the percentage (%) of torque, considering the initial torque of 100%. The comparison was performed within each group and between groups with and without mechanical fatigue (Table 3).

Significant differences were found among the three groups in the loosening test when analyzing the values with and without fatigue (*p* < 0.001) within each group. When the groups were compared with each other, there was also a significant difference between them (*p* < 0.001), except between groups GM and CMt without fatigue (*p* = 0.840) (Table 3). In the CMt group pull-out test, there was no mechanical frictional lock after initial loosening (without fatigue); however, it presented a mean value of 94.2 ± 12.82 N after fatigue.

**Figure 6:**
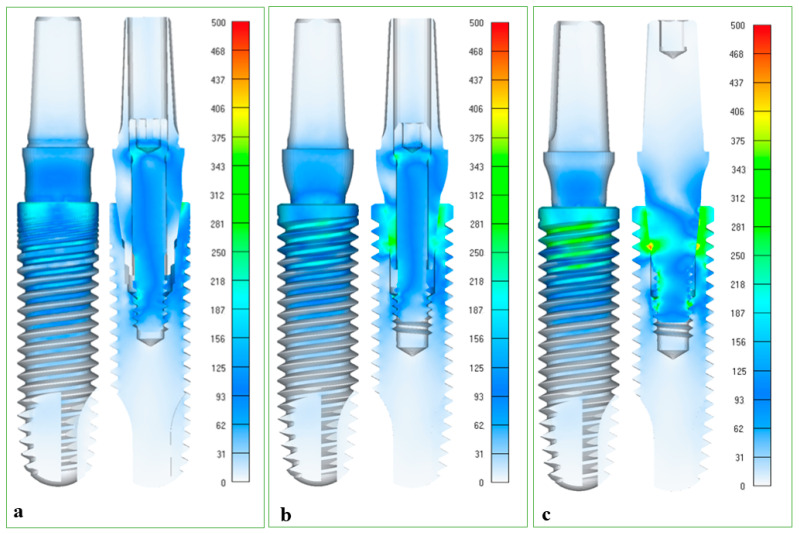
(**a**) Finite element analysis results of the stress behavior in the prosthetic abutment, in the upper third and middle third region of the implant, and screw preload for the 20 Ncm tightening torque under a 50 N load (GM group); (**b**) 15 Ncm tightening torque under a 50 N load (CMt group); (**c**) 32 Ncm tightening torque under a 50 N load (CMo group).

The FEA analysis revealed that the stress distribution varied among the three groups. When applying the load to the angled tested implant–abutment assemblies, the stress was found to be more concentrated in the upper third and middle third regions of the implant, as well as in the opposite region of the load application for all three groups. In the CMt and CMo groups, the stress concentration was also observed at the same end of the load application. However, the stress concentration was higher in the CMo group, where additional stress concentration regions near the implant–abutment tapered contact area were also observed (Figure 6).

The maximum von Mises stress results found for the groups are presented in Table 4.

The upper third region of the implant region of the body of the implants presented the highest stress values found in the three groups; however, GM and CMo presented higher values.

## 4. Discussion

The null hypotheses for screw loosening and stress level were rejected. There were differences in the values of loosening with and without fatigue and the stress level in the three groups studied.

In the abutment screw loosening test, percentages were used to measure the amount of torque loss or gain comparison between groups since the abutment tightening torque values indicated by the manufacturer are different (15 Ncm to 32 Ncm). Prior to the fatigue test, tightening torques were applied, and the loosening was measured after 5 min to serve as an evaluation parameter of a possible influence of fatigue on loosening torques [24]. According to the literature, the higher the number of fatigue cycles, the greater the initial torque loss value [24]. The fatigue test was performed at a frequency of 15 Hz, and a worst-case condition was simulated, leaving the 3 mm from the implant nominal bone level exposed [25]. These values are in accordance with ISO 14801 [15,19,20,25]. The number of cycles at each load was set at 5 × 10^6^ to mimic chewing conditions over a period of five to six years [16,19,20,25]. 

Failures in the implant body are relevant and concerning; these failures occur due to overloading, which is usually preceded by loosening of the screws, fracture of prostheses, or abutment screws, and yet it is associated with severe overload [26]. In the current study, the reduction in torque of abutments before fatigue (7.8% for GM and 9.67% for CMt) is within acceptable limits according to another study that reports that the deformation and flow of components can reduce torque applied at 2% to 10% in the first seconds or minutes after tightening [3]. Torque loss after the mechanical cycle can be caused by micro displacements of the prosthetic components when the screw interface is subjected to external loads. After this stage of “sedimentation effect” or relaxation, surface microroughness of the components may be present, and when micro displacements occur due to load incidence, there is a decrease in these surfaces of the components, which results in a decrease of retention screw preload [25].

The loosening test showed higher percentages of torque after fatigue for one-piece abutments of the CMo group (87.34%), while the two-piece abutments of the CMt group had the lowest values (49.19%); however, CMt abutments have mechanical frictional locks believed to help increase stability [24]. One study reported a torque percentage of 94.6% for one-piece abutments and 70% for two-piece abutments; however, the applied load simulated corresponded to three to four days of chewing function [24], while the current study corresponded to five to six years.

There are a few previous studies about GM system implants, which have been on the market for a short time with a different proposal for resistance improvement. These implants have a 16 degrees taper angle of the internally tapered portion (half-angle of 8) and self-removal of prosthetic components, which facilitates abutment removal in cases of a possible fracture of this component or prosthesis. The loosening of these abutments was high without fatigue (92.2%) and decreased after fatigue (67.2%). Past studies suggest that the angle of the implant’s tapered surface influences the reduction of loosening and fracture of screws [21,27]. The Morse taper system essentially creates a friction lock [21]. The tapered surface joined to another at an angle of fewer than 16 degrees creates a friction fit that closes mechanically; this adjustment decreases or increases depending on the respective angle changes [6]. In the current study, it was observed that the GM with greater angulation, even without frictional lock, maintained the lowest torque value after fatigue and still presented high stress in the FEA. Meanwhile, the CM with smaller angulation presented greater frictional lock in the pull-out test of the CMt group and in the FEA analysis, which shows a strong connection union and less stress. The three implant connections studied showed different behaviors but with good results, and the GM system, which is new on the market, showed promising results. However, animal and human studies are still needed to prove the system’s effectiveness. 

The tapered connections present mechanical characteristics that must be observed. The screw, the tapered abutment portions, and the implant act together in the process of insertion and removal of the abutments, both in one-piece and two-piece abutments [1]. The engagement at the implant–abutment junction occurs through the frictional resistance resulting from contact between the tapered coupling sections, and the bolt only helps to guide positioning [1]. In this study, it was not possible to perform the pull-out test in the CMo group because the abutments are one-piece or for the GM group abutments, which have a component self-removal system. However, for the two-piece abutments of the CMt group, it was possible to loosen the screws and evaluate the pull-out.

The pull-out test is one of the methods used to evaluate the implant–abutment stability [16]. In this current study, the average pull-out value was used for the CMt group (11.5 degrees taper angle), the implant used was 3.75 mm in diameter, and the value presented was 94.2 N. In another study, the same type of implant of 3.75 mm in diameter was used (11.5 degrees taper angle), and mean pull-out values ranged from 27 to 49.6 N [16]. The abutments used in the two studies had different diameters and manufacturing characteristics. The abutment used in the current study has a larger diameter in the cementable part, with a larger load-receiving area, which may explain the higher pull-out values in the previous study. This test can be used to show whether the results found in the loosening values are a clinical risk or not. Even though there is a higher percentage of loosening of the screw, the mechanical lock can guarantee that the abutment will not come loose, keeping the prosthesis in the mouth.

The selection of a 50 N load for FEA in the mechanical test for all groups in this study was justified based on its sufficiency in evaluating the stress distribution. This load value was chosen considering previous research [13,22] and its ability to provide a detailed view of the mechanics inherent in the simulated system. The stress concentration was different for the three groups, regardless of the simulated preload, and the FEA showed that the stress was more concentrated in the upper third and middle third regions of the implant of the CMt and CMo implants. However, in the abutments, the stress concentration was in the upper third region of the CMt and CMo abutments on the load application side and on the screw of the CMo abutments, especially in the threaded region. This behavior suggests that despite the tapered interlock between the tapered surfaces, the screw region still contributes to mechanical stability [13,21]. This mechanism protects the abutment threads from excessive cyclic functional loading and provides stable retention [21].

The results of the von Mises maximum stress in the upper third region of the implant body were different such that the GM and CMt groups presented higher stress concentration values compared to CMo (Table 4) (Figure 6), possibly due to the simulated force characteristics in inclined specimens. Increased stresses were also found in the middle of the abutment and implant body in the tapered contact region. Another study used the same implant system (Morse taper 3.5 mm) with a similar one-piece abutment and had a lower von Mises stress than presented here, regardless of the preloads applied (200 N and 320 N) [13]. This greater stress can be explained by the 30 degrees of inclination of the specimens, while in the previous study, 15 degrees of inclination were used. The smaller deformation (von Misses) that occurred in the CMo group may explain why the abutments in this group had the greatest loosening before and after fatigue.

In a previous study, it is suggested to test the prosthetic abutments of one-piece and two-piece from a single manufacturer to compare the torques [13]. In the current study, it was confirmed that the loosening is more stable in the abutment of one-piece compared to two-piece abutments. The design of the internal tapered connection previously showed less torque loss compared to the external connections; the use of coated screws also led to higher torque maintenance compared to uncoated titanium screws [2]. Further studies could test other implant designs and manufacturers, different internal tapers, and implant–abutment connection designs that remain quite commercialized. Clinical studies could also test the stability of different implant–abutment connections using different torque levels, as was performed in a previous study [13], and perform bone level measurements in these connections.

Under the limitations of this experimental in vitro and FEA study, the maximum force supported was influenced by oblique cyclic loading in the implant–abutment assemblies of the Morse taper systems but was not the Grand Morse system. The stress behavior (FEA) was different for three groups, in the upper third and middle third regions in GM and GMt, and for the abutments, the implant–abutment tapered contact region, body, and screw threads (CMo) presented higher stress concentration.

## 5. Conclusions

Although the CMo group showed lower rates of loosening, it displayed a poorer stress distribution in comparison to the GM and CMt groups. On the other hand, the CMt group exhibited a satisfactory frictional lock after undergoing the fatigue tests.

## Figures and Tables

**Table 1:** Test groups and conditions.

Group	Implant	Abutment	Connection Type/Index
GM	Titamax GM Neodent Ø3.75/13 mm	Universal Abutment GM exact Neodent Ø3.3/6/2.5 mm (two-piece)	Internal Tapered/Internal Hexagon/16 degrees
Mt	Titamax Cortical CM Neodent Ø3.75/13 mm	Universal Abutment CM Neodent through screw Ø3.3/6/2.5 mm (two-piece)	Internal Tapered/ Without Index/11.5 degrees
CMo	Titamax Cortical CM Neodent Ø3.75/13 mm	Universal Abutment CM Neodent Ø3.3/6/2.5 mm (one-piece)	Internal Tapered/Without Index/11.5 degrees

**Table 2:** Material parameters used for the numerical analyses provided by the manufacturer.

Material	E (GPa)	Poisson’s Ratio
Implants	103	0.361
Abutments	105	0.361
Hemispheric body	105	0.361
Polyacetal	3.1	0.3

**Table 3:** Statistical comparison of torque loss (%) with and without mechanical fatigue in each group.

	GM	CMt	CMo
Without mechanical fatigue	92.2 (2.54) Aa	90.33 (6.09) Aa	−110.74 * (9.92) Ab
With mechanical fatigue	67.2 (3.46) Bc	49.19 (12.14) Bb	87.34 (5.37) Ba

Different letters show statistical differences in the upper case (intra groups, vertical) and lower case (between groups, horizontal) (*p* < 0.05). Comparison of torque loss (%) without and with mechanical fatigue within each implant–abutment group—two-piece abutment (GM and CMt Groups) and one-piece (CMo Group); * Torque gain.

**Table 4:** Maximum von Mises stress values (MPa) after simulated force application.

Groups	Simulated Force (50 N)
GM	243 MPa
CMt	222 Mpa
CMo	250 MPa

## Data Availability

The datasets generated and analyzed during the current study are available from the corresponding author upon reasonable request.
